# Supplementary material for: Multi‐dataset identification of innovative feature genes and molecular mechanisms in keratoconus
Source: J Cell Mol Med. 2024 Sep 19;28(18):e70079. doi: 10.1111/jcmm.70079 (PMC11412914; doi:10.1111/jcmm.70079)
Supplement: Supplementary file 2 — Material S2. [file JCMM-28-e70079-s002.zip › 7.GSEA analysis/5.RNF217-AS1/KEGG_SELENOAMINO_ACID_METABOLISM.html]

Details for gene set KEGG\_SELENOAMINO\_ACID\_METABOLISM[GSEA]

|  || Dataset | RNF217-AS1.group.cls#h\_versus\_l |
| Phenotype | group.cls#h\_versus\_l |
| Upregulated in class | l |
| GeneSet | KEGG\_SELENOAMINO\_ACID\_METABOLISM |
| Enrichment Score (ES) | -0.5014769 |
| Normalized Enrichment Score (NES) | -1.4735228 |
| Nominal p-value | 0.06762295 |
| FDR q-value | 0.30995175 |
| FWER p-Value | 0.8 |
Table: GSEA Results Summary

  

Fig 1: Enrichment plot: KEGG\_SELENOAMINO\_ACID\_METABOLISM      
 Profile of the Running ES Score & Positions of GeneSet Members on the Rank Ordered List

  

| SYMBOL | TITLE | RANK IN GENE LIST | RANK METRIC SCORE | RUNNING ES | CORE ENRICHMENT || 1 | TRMT11 | na | 10210 | 0.083 | -0.2557 | No |
| 2 | GGT6 | na | 11366 | 0.065 | -0.2589 | No |
| 3 | AHCY | na | 13659 | 0.034 | -0.3090 | No |
| 4 | MAT2A | na | 13771 | 0.033 | -0.2968 | No |
| 5 | PAPSS1 | na | 13952 | 0.031 | -0.2875 | No |
| 6 | GGT7 | na | 14223 | 0.028 | -0.2822 | No |
| 7 | METTL2B | na | 15371 | 0.015 | -0.3082 | No |
| 8 | GGT1 | na | 16634 | 0.002 | -0.3434 | No |
| 9 | LCMT1 | na | 17666 | -0.008 | -0.3695 | No |
| 10 | AHCYL1 | na | 18071 | -0.011 | -0.3758 | No |
| 11 | MAT2B | na | 18082 | -0.012 | -0.3708 | No |
| 12 | MAT1A | na | 18213 | -0.013 | -0.3686 | No |
| 13 | SEPHS2 | na | 19720 | -0.027 | -0.3993 | No |
| 14 | SEPHS1 | na | 20082 | -0.031 | -0.3954 | No |
| 15 | CBS | na | 23765 | -0.072 | -0.4681 | Yes |
| 16 | LCMT2 | na | 24680 | -0.086 | -0.4547 | Yes |
| 17 | CTH | na | 25070 | -0.091 | -0.4235 | Yes |
| 18 | METTL6 | na | 26062 | -0.108 | -0.4018 | Yes |
| 19 | SCLY | na | 27148 | -0.129 | -0.3731 | Yes |
| 20 | HEMK1 | na | 27571 | -0.138 | -0.3210 | Yes |
| 21 | AHCYL2 | na | 28262 | -0.155 | -0.2688 | Yes |
| 22 | GGT5 | na | 29647 | -0.195 | -0.2180 | Yes |
| 23 | MARS2 | na | 32383 | -0.316 | -0.1502 | Yes |
| 24 | PAPSS2 | na | 33931 | -0.469 | 0.0230 | Yes |
Table: GSEA details [plain text format]

  

Fig 2: KEGG\_SELENOAMINO\_ACID\_METABOLISM      
 Blue-Pink O' Gram in the Space of the Analyzed GeneSet

  

Fig 3: KEGG\_SELENOAMINO\_ACID\_METABOLISM: Random ES distribution      
 Gene set null distribution of ES for **KEGG\_SELENOAMINO\_ACID\_METABOLISM**
